# Transcriptome analysis reveals tumor antigen and immune subtypes of melanoma

**DOI:** 10.32604/or.2023.029274

**Published:** 2023-05-24

**Authors:** JIAHENG XIE, MENGMENG OU, PAN YU, DAN WU, QIKAI TANG, YUAN CAO, JING HANG, LU YIN, TINGHONG XIANG, MING WANG, JINGPING SHI

**Affiliations:** 1 Department of Burn and Plastic Surgery, The First Affiliated Hospital of Nanjing Medical University, Jiangsu Province Hospital, Nanjing, 210029, China; 2 Department of Rheumatology and Immunology, Nanjing Drum Tower Hospital, The Affiliated Hospital of Nanjing University Medical School, Nanjing, 210008, China; 3 Department of Neurosurgery, The First Affiliated Hospital of Nanjing Medical University, Jiangsu Province Hospital, Nanjing, 210029, China; 4 Fourth School of Clinical Medicine, Nanjing Medical University, Nanjing, 210029, China; 5 Department of Ultrasound in Medicine, The First Affiliated Hospital of Nanjing Medical University, Jiangsu Province Hospital, Nanjing, 210029, China

**Keywords:** Melanoma, mRNA vaccine, Immune microenvironment, Immunotherapy, Bioinformatics analysis

## Abstract

**Purpose:**

To screen potential tumor antigens for melanoma vaccine development and identify different immune subtypes.

**Methods:**

Transcriptional data (HTSEQ-FPKM) and clinical information of a 472 Melanoma cohort GDC TCGA Melanoma (SKCM) were downloaded from the UCSC XENA website (http://xena.ucsc.edu/). Subsequently, transcriptome data and clinical information of 210 melanoma cohort GSE65904 were downloaded from Gene Expression Omnibus (GEO), a large global public database. All the transcriptome expression data matrices were log2 transformed for subsequent analysis. GEPIA, TIMER, and IMMPORT databases are also used for analysis. Cell function experiments were performed to validate the role of the IDO1 gene in melanoma cell line A375.

**Results:**

Our study provides potential tumor antigens for vaccine development in melanoma patients: GZMB, GBP4, CD79A, APOBEC3F, IDO1, JCHAIN, LAG3, PLA2G2D, XCL2. In addition, we divide melanoma patients into two immune subtypes that have significant differences in tumor immunity and may have different responses to vaccination. In view of the unclear role of IDO1 in melanoma, we selected IDO1 for cell assay validation. Cell function assay showed that IDO1 was significantly overexpressed in the melanoma A375 cell line. After IDO1 knockdown, the activity, invasion, migration and healing ability of A375 cell lines were significantly decreased.

**Conclusion:**

Our study could provide a reference for the development of vaccines for melanoma patients.

## Introduction

The discovery of vaccines was a milestone in the history of human medicine [1]. It originated in the 18th century when Edward Jenner discovered that vaccinia protected against smallpox, opening the door to the development of a vaccine [2]. Since then, vaccines have gained much attention for their ability to prevent and treat diseases, especially infectious ones [3]. The current outbreak of COVID-19 has made people aware of the importance of vaccine development, especially mRNA vaccines [4]. It is worth mentioning that the application of mRNA vaccines is not limited to infectious diseases, and breakthrough progress has been made in the development of mRNA vaccines for some specific cancer types [5]. tumor mutation load, microsatellite instability, and changes in tumor microenvironment all provide clues for the development of mRNA vaccine [6]. It is of great clinical significance to explore the immune microenvironment of tumors and screen potential mRNA vaccines. The original mRNA vaccines had some disadvantages such as instability, excessive immunogenicity, and lack of delivery system [7]. However, as the technology improves, mRNA vaccines are emerging as an effective and stable new treatment option for cancer and viral diseases. Now mRNA vaccines are becoming more attractive.

Melanoma is a kind of malignant tumor highly related to immunity [8]. Current immunotherapies for melanoma, such as immune checkpoint inhibitors, have achieved breakthrough results [9]. But unfortunately, there are still a considerable number of patients with a low response or drug resistance, resulting in disease recurrence and metastasis [10]. Therefore, our understanding of the immune microenvironment of melanoma is still inadequate [11]. However, the advent of RNA vaccines has opened the door to treatments for refractory melanoma. Sahin et al. found that FixVac (BNT111), an intravenous liposomal RNA vaccine, induced strong and persistent CD4+ and CD8+ T cell immunity [12]. Salotto et al. constructed a melanoma cancer vaccine based on nano liposome and found that it was effective in activating CD4+ T cells through *in vitro* and *in vivo* experiments [13]. Therefore, the RNA vaccine may be the most promising treatment for melanoma in the future.

In this study, mutation analysis, immune cell infiltration analysis, survival analysis, single-cell analysis, weighted co-expression network analysis, and immune microenvironment analysis were performed by combining melanoma data from multiple tumor databases. Our study may provide some new ideas for the diagnosis and treatment of melanoma.

## Materials and Methods

### Data sources and processing

First, transcriptional data (HTSEQ-FPKM) and clinical information of a melanoma cohort of 472 patients called GDC TCGA Melanoma (SKCM) were downloaded from the UCSC XENA website (http://xena.ucsc.edu/). Subsequently, transcriptome data and clinical information of 210 melanoma cohort GSE65904 were downloaded from Gene Expression Omnibus (GEO), a large global public database. All the transcriptome expression data matrices were log2 transformed for subsequent analysis.

### cBioportal database

cBioportal website (http://www.cbioportal.org/) is an open and comprehensive web tool that be used to explore the tumors data [12]. In this study, to ensure data consistency, a skin cutaneous melanoma cohort (TCGA, Firehose Legacy) was selected in this study, which included 479 samples. Then through the analysis function of the website, the copy number changes and mutations of genes in melanoma were obtained. We then picked out the genes that had both copy-number amplification and mutations.

### GEPIA2 database

GEPIA2 is a comprehensive analysis website based mainly on TCGA data [13]. In this study, by setting the dataset for SKCM, |log2FC| Cutoff is 1, the *q*-value Cutoff is 0.01, we conducted between melanoma and normal tissue differences in gene analysis. After the differentially expressed genes were obtained, the up-regulated genes were retained for subsequent analysis. Possible tumor antigens can be obtained by crossing the upregulated genes with the previously obtained genes that are both mutated and amplified. Then, through the survival analysis function of this website, the samples were divided into high expression and low expression groups with the median of gene expression as the truncation value, and the direct differences in overall survival and disease progression-free (DFS) between the two groups were evaluated using log-rank test, and *p* < 0.05 was defined as statistically significant differences.

### TIMER database

The TIMER database (https://cistrome.shinyapps.io/timer/) is a web site can be used to explore immune cells infiltration. In this study, the site’s Gene module was used to explore the association of genes with immune cell infiltration in melanoma by setting the cancer type to SKCM.

### IMMPORT database

IMMORT is a web site that explores the relationship between cancer and immunity. In this study, 2483 immune-related genes were downloaded from the “Shared Data” section of the website.

### Unsupervised cluster analysis

We first identified immune-related genes with prognostic value in melanoma by univariate COX analysis. Then, based on these genes, an unsupervised cluster analysis was performed using the R-package “ConsensusClusterPlus”. The parameters set are: maximum K value is 6, pItem is 0.8, REPS is 1000, clusterAlg is “PAM”, distance is set to “Euclidean”. Then, the optimal clustering number is determined according to PAC’s method.

### Mutant landscape analysis

We used the “oncoplot” function of R package “maftools” to show the mutation of the top 20 genes with the highest mutation frequency in different immune subtypes. The tumor mutation load (TMB) for each sample was then calculated using the TMB function.

### Immune correlation analysis

We first reviewed the literature to summarize 47 immune checkpoint genes and 25 immunogenic cell death (ICD) regulatory genes. Then the expression of these genes in different immune subtypes was obtained by differential analysis. Subsequently, 28 marker genes of immune cells were collected through literature review, and the immune cell score of each sample was calculated by R package “ssGSEA”. In order to explore the differences in the distribution of immune cells in different immune subtypes, heat maps and bar charts were used to show the results respectively. We then calculated the immune score, stromal score, total score, and tumor purity score for each sample using the R package “Essclause”, and compared the differences in these scores among different immune subtypes. Finally, we used the R package “ImmuneSubtypeClassifier” to divide melanoma patients into different subtypes (C1–C6) and explore the correspondence between different immune subtypes.

### Immune landscape analysis

We used the reduceDimension function of R package “Monocle” to conduct dimension reduction analysis, set max_components to 2, and use the method “DDRTree” to get different states of cells. Then the plot_cell_trajectory function is used to display the results of different immune subtypes.

### Gene co-expression analysis

WGCNA (Weighted Gene co-expression network analysis) is an analysis method designed to find co-expressed gene modules by calculating the weight between genes. In this study, by WGCNA analysis of the previously obtained immune genes, the optimal soft threshold value was set as 5, the minimum number of module genes was set as 30, deepSplit was set as 3, and modules with MEDissThres lower than 0.3 were merged, finally, 5 modules were obtained. The differences in the characteristics of these modules in different immune subtypes were then explored.

### Cell lines, culture conditions and cell transfection

Human melanoma A375 cells were purchased from the Chinese Cell Repository (Shanghai, China). Human immortalized keratinocytes (HaCaT) were purchased from the Cell Resource Center of Peking Union Medical College, China. All cells were cultured in high-glucose DMEM (Gibco), containing 10% fetal bovine serum (FBS, Gibco) and 1% penicillin-streptomycin solution (Gibco) at 37°C with 95% air and 5% CO_2_ with saturated humidity. Transfection of cells with siRNAs (Suppl. Table S1) was carried out using lipofactamine3000 (Thermo Fisher Scientific, Waltham, MA, USA) according to the manufacturer’s instructions.

### Quantitative real‑time polymerase chain reaction (qRT-PCR)

The total RNA was extracted from cultured cells with Trizol reagent (Invitrogen) and reverse-transcribed with PrimeScript RT Reagent Kit with gDNA Eraser (TaKaRa). qRT-PCR was implemented utilizing AceQ Universal SYBR qPCR Master Mix (Vazyme, Nanjing, China) on an ABI Stepone plus PCR system (Applied Biosystems, FosterCity, CA, USA). The sequences of primers were listed in Suppl. Table S2. Relative quantification was determined using the 2^−ΔΔCt^ method.

### CCK-8 assay

The Cell Counting Kit-8 (CCK8) method was used to assess cell proliferation. The cells were seeded in a 96-well cell culture plate. Subsequently, 24 hours after siRNA transfection, each day of the following 4 days. 10 μL of CCK-8 solution (Dojindo, Japan) were added to each well of the plate. After incubating for 2 h at 37°C away from light, Finally, the absorbance of each well was detected with a microplate reader at 450 nm.

### Colony formation analysis

The transfected A375 cells were kept in 6-well plates for approximately 14 days. Thereafter, the clones were stained with crystal violet and then imaged and counted.

### Wound healing, migration, and invasion assays

Wound healing, transwell migration, and invasion assays were performed as previously reported by us [14].

### Statistical analysis

Experimental data were processed using the SPSS software (version 26.0). All data were illustrated employing mean ± SD. One-way Analysis of Variance (ANOVA) and Turkey’s multiple comparisons of the means were employed in the analysis of multiple sets of data unless otherwise specified.

## Results

### Preliminary screening of possible tumor antigens in melanoma

**Figure 1:**
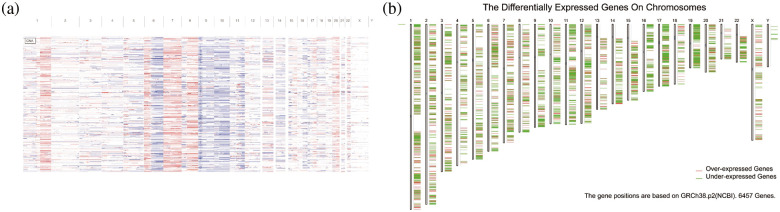
Mutation analysis. (a) Copy number variation. (b) Differentially expressed genes on chromosomes.

Copy-number analysis of genes in melanoma showed that there were more copy-number variations in melanoma patients, which may be a factor for the poor prognosis of melanoma (Fig. 1a). Furthermore, 18,193 amplified genes were screened. After a further screening of mutated genes, a total of 13,914 mutated and amplified genes were obtained. Next, as shown in Fig. 1b, we found that through differential expression analysis, a total of 6457 differentially expressed genes were obtained in melanoma according to screening criteria, among which 2546 genes were highly expressed in cancer. Finally, by taking intersection, we obtained a total of 1489 genes with both mutation, copy-number amplification, and high expression in melanoma, which were selected as tumor antigen genes obtained by preliminary screening for subsequent analysis.

### Prognostic analysis and immune cell infiltration analysis of possible tumor antigens

**Figure 2:**
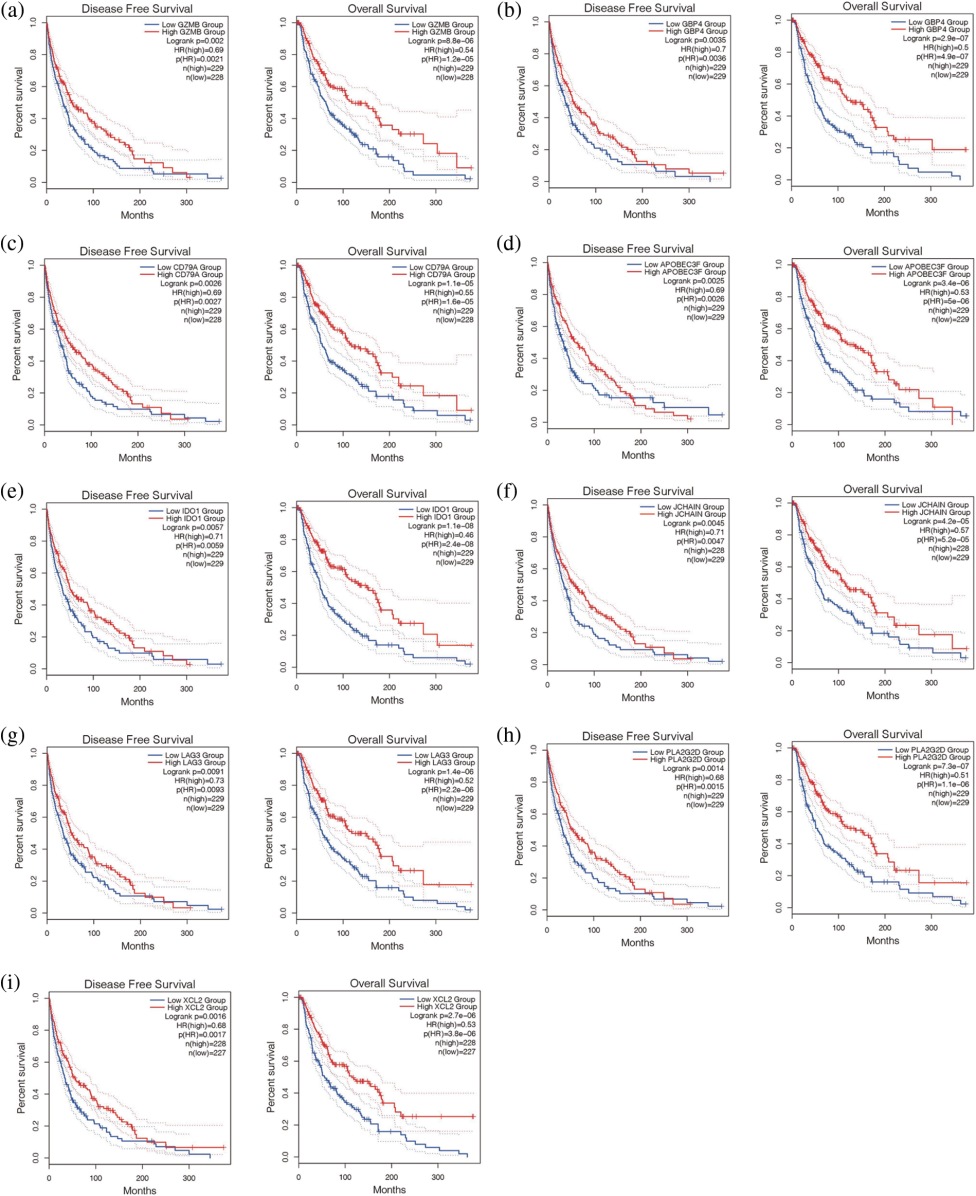
Survival analysis of potential tumor antigens. Disease-free survival is shown on the left and overall survival is shown on the right. (a–i) Nine antigen genes were screened out of 1489 possible tumor antigens, which were APOBEC3F, CD79A, GBP4, GEMB, IDO1, JCHAIN, LAG3, PLA2G2D, and XCL2.

Finally, nine antigen genes were screened out of 1489 possible tumor antigens, which were APOBEC3F, CD79A, GBP4, GZMB, IDO1, JCHAIN, LAG3, PLA2G2D, and XCL2 (Figs. 2a–2i). They were not only related to the overall survival (OS) but also related to the disease-free progression survival (RFS) (*p* < 0.05). At the same time. Their upregulation was associated with a better prognosis.

**Figure 3:**
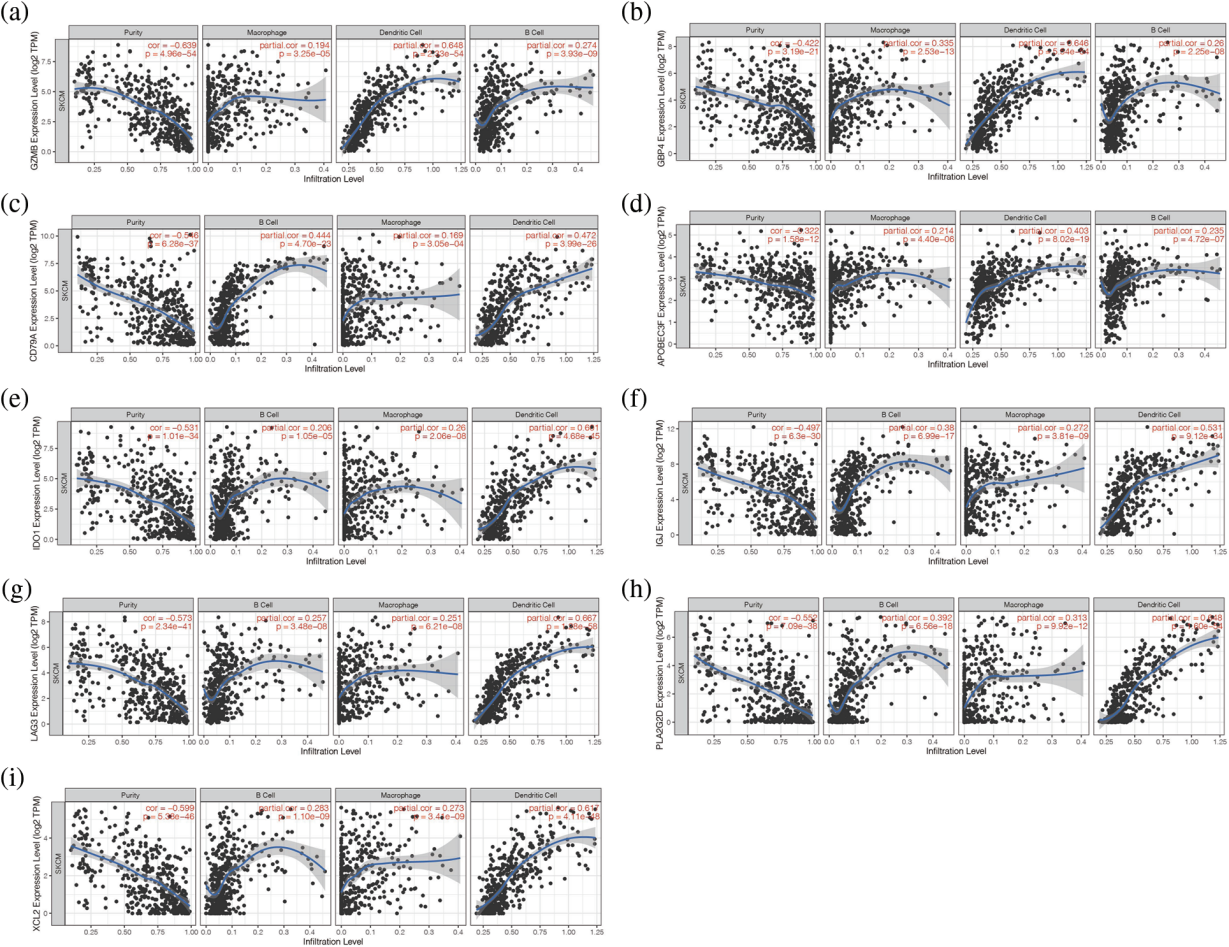
Analysis of immune microenvironment associated with these 9 genes. (a–i) The nine genes were all correlated with macrophage cells, dendritic cells, and B cells.

In addition, we found that the nine genes were all correlated with macrophage cells, dendritic cells, and B cells (Figs. 3a–3i). Then, the expression of these nine genes was negatively correlated with tumor purity (*p* < 0.05, Figs. 3a–3i). It is suggested that these nine genes may be tumor antigen genes of melanoma. Next, we screened melanoma populations suitable for vaccination.

### Unsupervised cluster analysis

**Figure 4:**
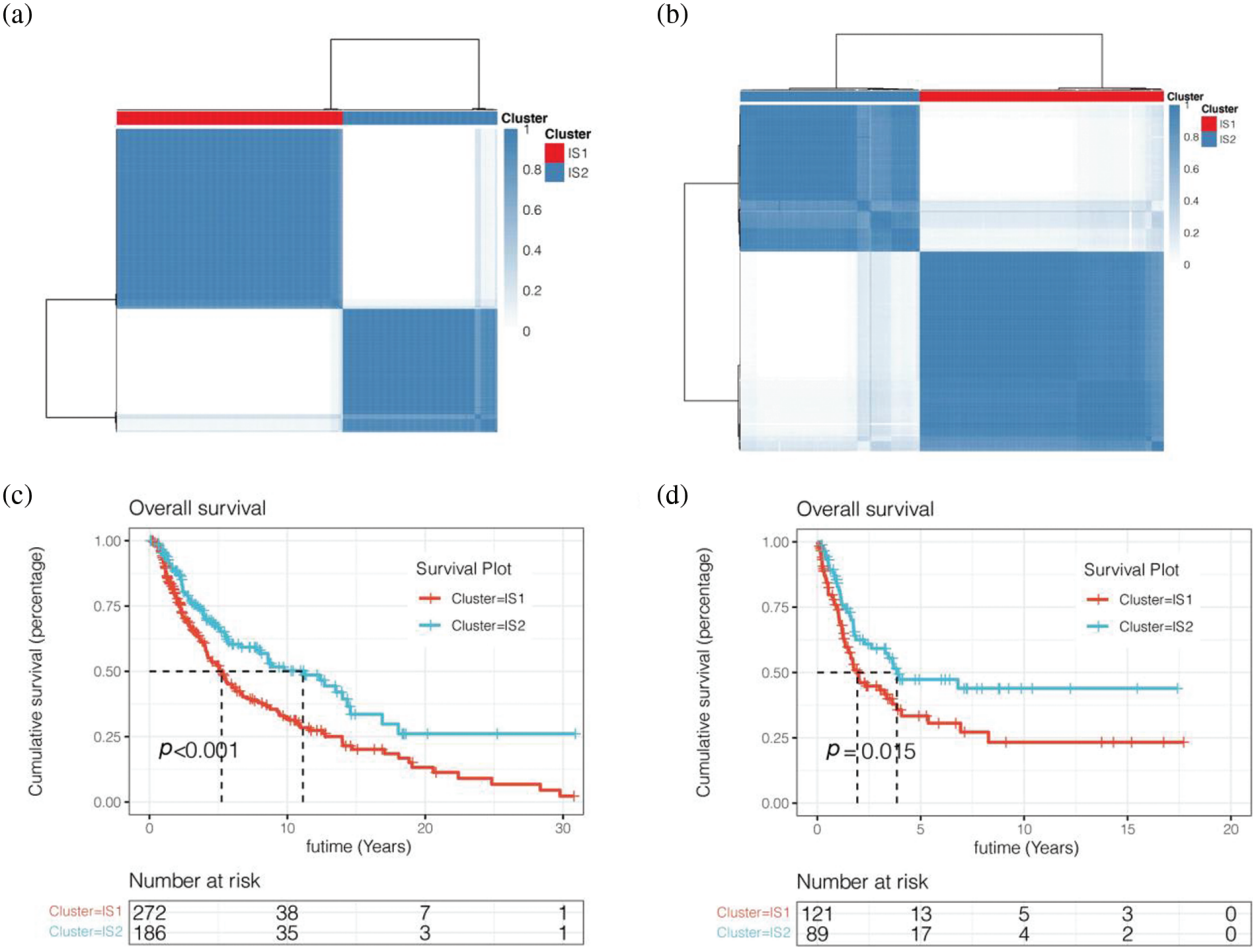
Unsupervised cluster analysis. (a) Two immune subtypes (IS1 and IS2) were obtained. (b) Tluster analysis in the GSE65904 cohort from the GEO database. (c) Survival analysis was performed for IS1 and IS2 subtypes of the TCGA cohort. (d) Survival analysis was performed for IS1 and IS2 subtypes of the GEO cohort.

We first downloaded 2483 immune-related genes from the IMMPORT database. Then we obtained prognostic immune-related genes by univariate COX analysis. We then clustered melanoma patients in the TCGA cohort by unsupervised clustering (Fig. 4a). According to the PCA method, two immune subtypes (IS1 and IS2) were obtained. We then analysed the prognosis of the two immune subtypes and found that the prognosis of IS2 patients was worse than that of IS1 patients, *p* < 0.001 (Fig. 4c). To further verify the reliability of the classification results, we also used the above method for cluster analysis in the GSE65904 cohort from the GEO database (Figs. 4b and 4d). The results showed that patients were also clustered into two groups, immune subtypes IS1 and IS2. By prognostic analysis of the two immune subtypes, it was also found that the prognosis of melanoma patients with the IS2 immune subtype was worse than that of patients with the IS1 immune subtype, *p* < 0.05 (Figs. 4b and 4d).

### Differences in mutant landscapes among immune subtypes

**Figure 5:**
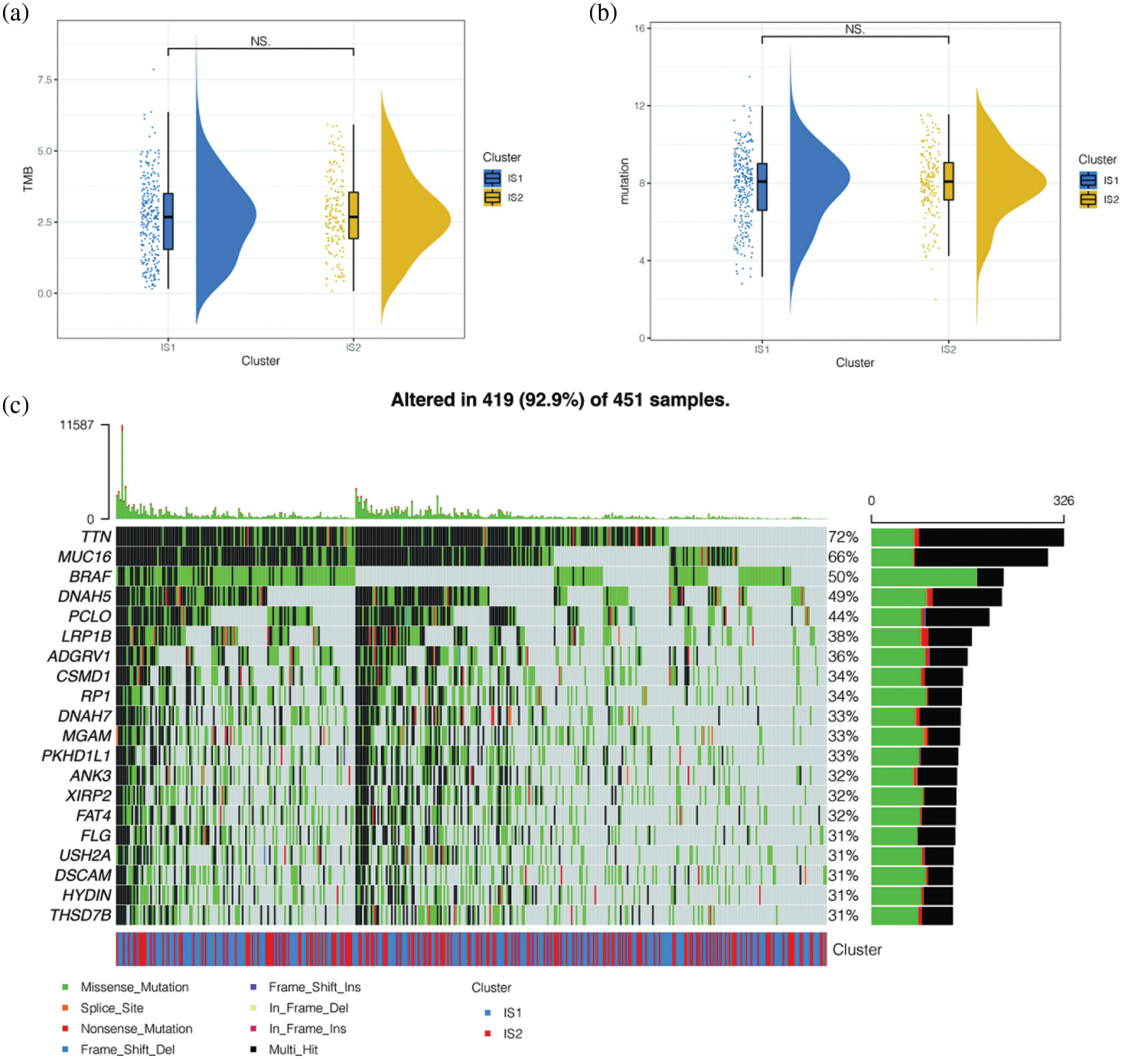
Differences in mutant landscapes among immune subtypes. (a) Tumor mutation load (TMB) and the total number of mutations per melanoma patient. No significant difference was found between immune subtypes IS1 and IS2 in TCGA cohort. (b) Tumor mutation load (TMB) and the total number of mutations per melanoma patient. No significant difference was found between immune subtypes IS1 and IS2 in GEO cohort. (c) The mutation of the first 20 genes in melanoma patients and their distribution in immune subtypes. The top 20 gene mutations were mutated in 419 (92.9%) samples. The more common types of mutations were deletion mutation and Multi_Hit, and the gene with the highest mutation frequency was TTN.

To further explore the differences in mutation landscape among immune subtypes, we calculated the tumor mutation load (TMB) and the total number of mutations per melanoma patient using the “mafTools” package. No significant difference was found between immune subtypes IS1 and IS2 (Figs. 5a and 5b). We then showed the mutation of the first 20 genes in melanoma patients and their distribution in immune subtypes (Fig. 5c). We found that among melanoma patients in the TCGA database, the top 20 gene mutations were mutated in 419 (92.9%) samples. The more common types of mutations were deletion mutation and Multi_Hit, and the gene with the highest mutation frequency was TTN. Many scholars have reported that TTN gene mutation is related to the occurrence and development of various cancers, and the main type of TTN mutation is Multi_Hit.

### Differences in immunity between two immune subtypes

**Figure 6:**
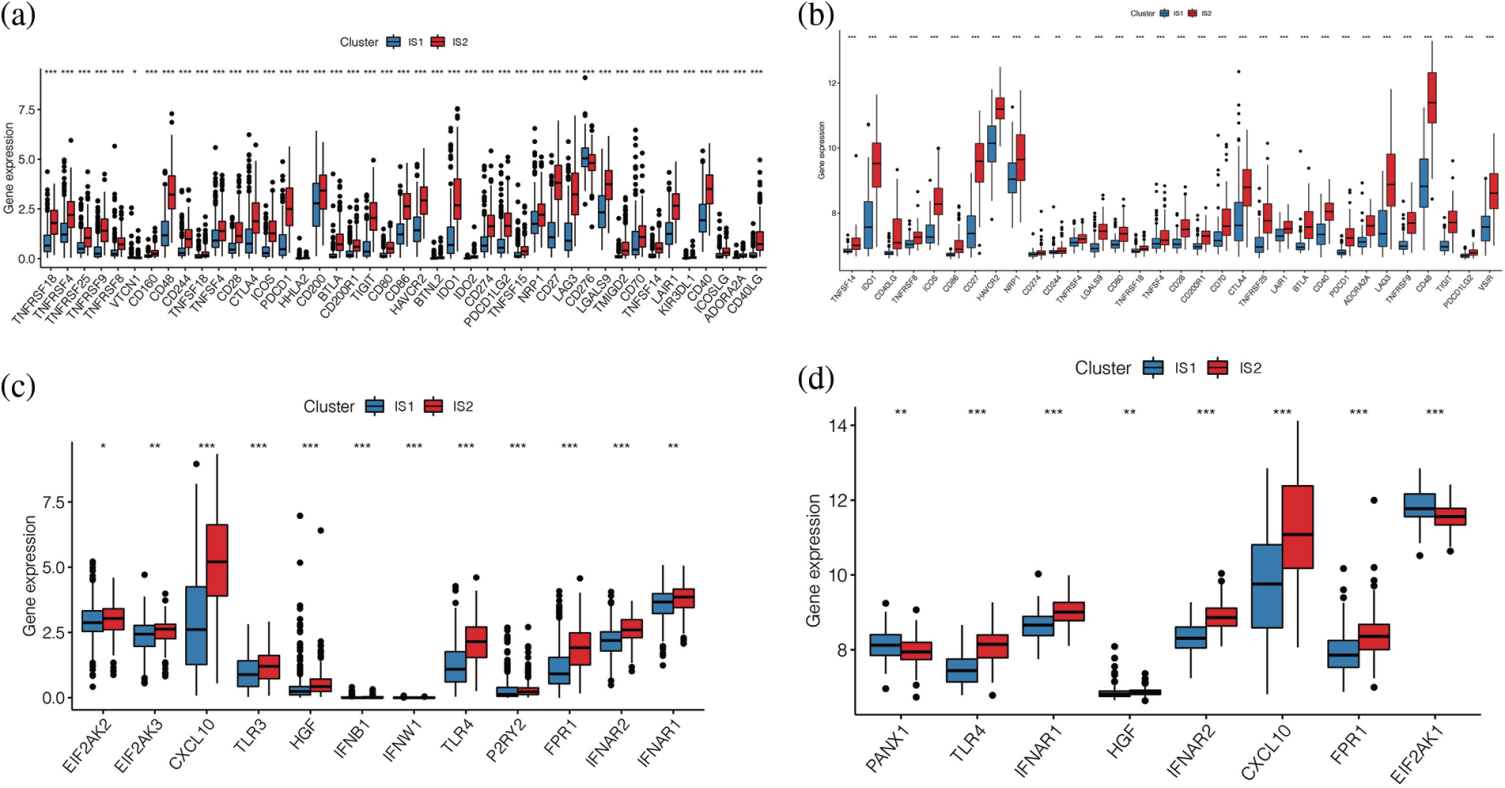
Differences in immunity between two immune subtypes. (a, b) Differential expression of immune checkpoint related genes in TCGA cohort and GEO cohort. (c, d) The distribution of immunogenic cell death (ICD) regulation genes in the two immune subtypes.

To further explore the immunological differences between these two immune subtypes, we analysed the differences in the expression of immune checkpoint genes and immunogenic cell death (ICD) regulation genes between the two immune subtypes. The results indicated that, as shown in Figs. 6a and 6b, most of the immune checkpoint genes were upregulated in immune subtype IS2, both in TCGA data and in the GSE65904 cohort (*p* < 0.05), including common CD274, CTLA4, PDCD1G2, etc. The distribution of immunogenic cell death (ICD) regulation genes was also different in the two immune subtypes (Figs. 6c and 6d), and it was found that ICD was highly expressed in the immune subtype IS2. Overall, both ICPs and ICDs are highly expressed in immune subtype IS2 in melanoma. Moreover, we found that the immune subtypes IS1 and IS2 can better distinguish the expression levels of immune checkpoint genes and ICDs.

### Immune cell infiltration analysis

**Figure 7:**
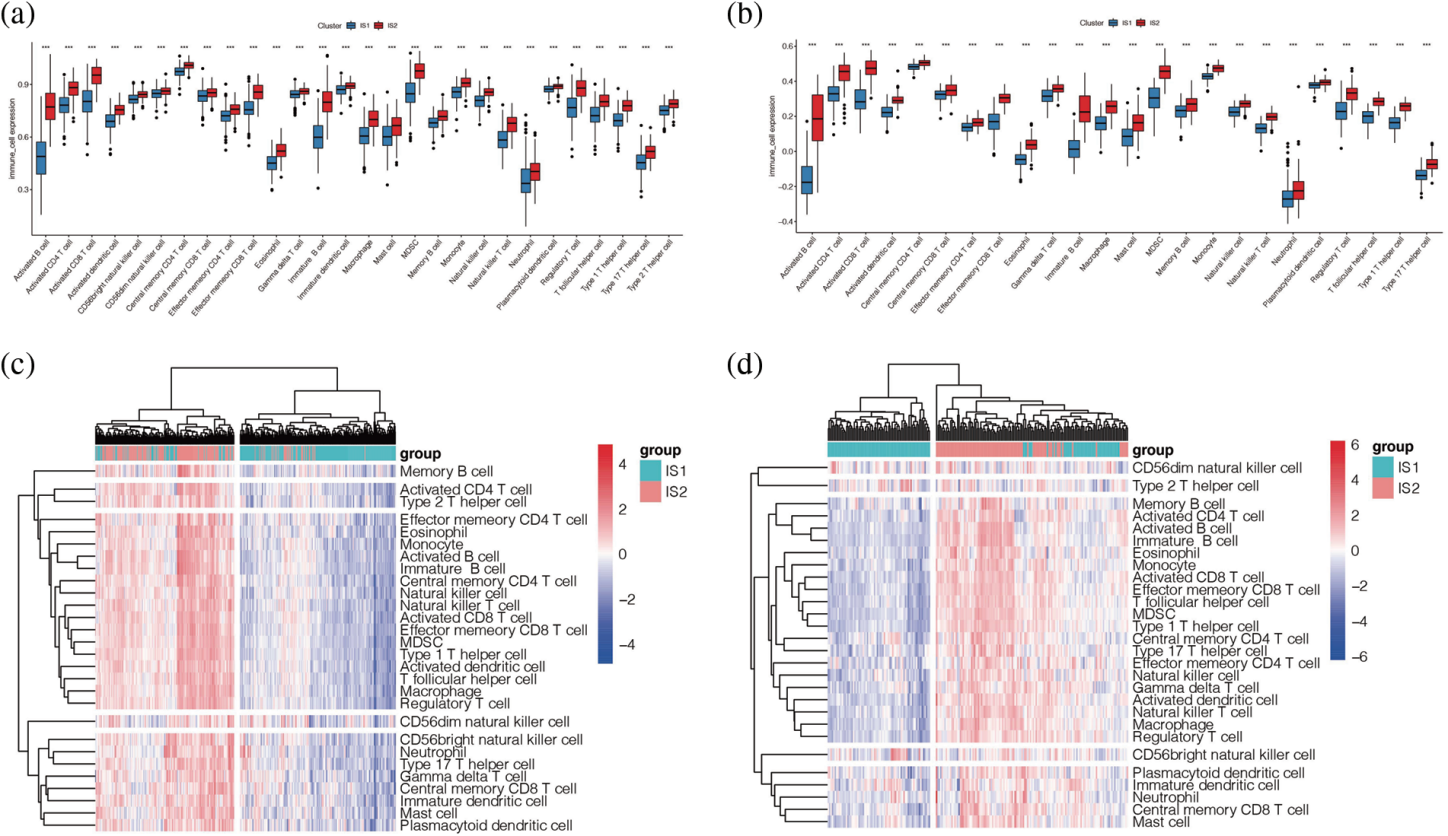
Immune cell infiltration analysis. (a, b) Enrichment of immune cells from melanoma patients in TCGA (a) and GEO (b) cohorts we found that in the two cohorts are immune cells in the immune subtypes IS2 score is high IS1, prompt immune cell activation and expression of IS2 may have more, including various T cells, B cells and so on. (c, d) Heat maps of immune cells from melanoma patients in TCGA (c) and GEO (d) cohorts.

Each patient’s immune cell score was quantified by ssGSEA analysis. To specifically distinguish which express differences between the two subtypes of immune cells are statistically significant, we explored the specific differences between them in immune subtype analysis, as shown in Figs. 7a and 7b. We found that in the two cohorts are immune cells in the immune subtypes IS2 with a high IS1 score, prompt immune cell activation and expression of IS2 which may have more, including various T cells, B cells and so on. These results suggest that the immune subtype IS2 may be the immune-hot type and IS1 may be the immune-cold type. As shown in Figs. 7c and 7d, immune cells in both cohorts were highly enriched in immune subtype IS2, and the darker the red colour was, the higher the score of immune cells in melanoma samples were.

**Figure 8:**
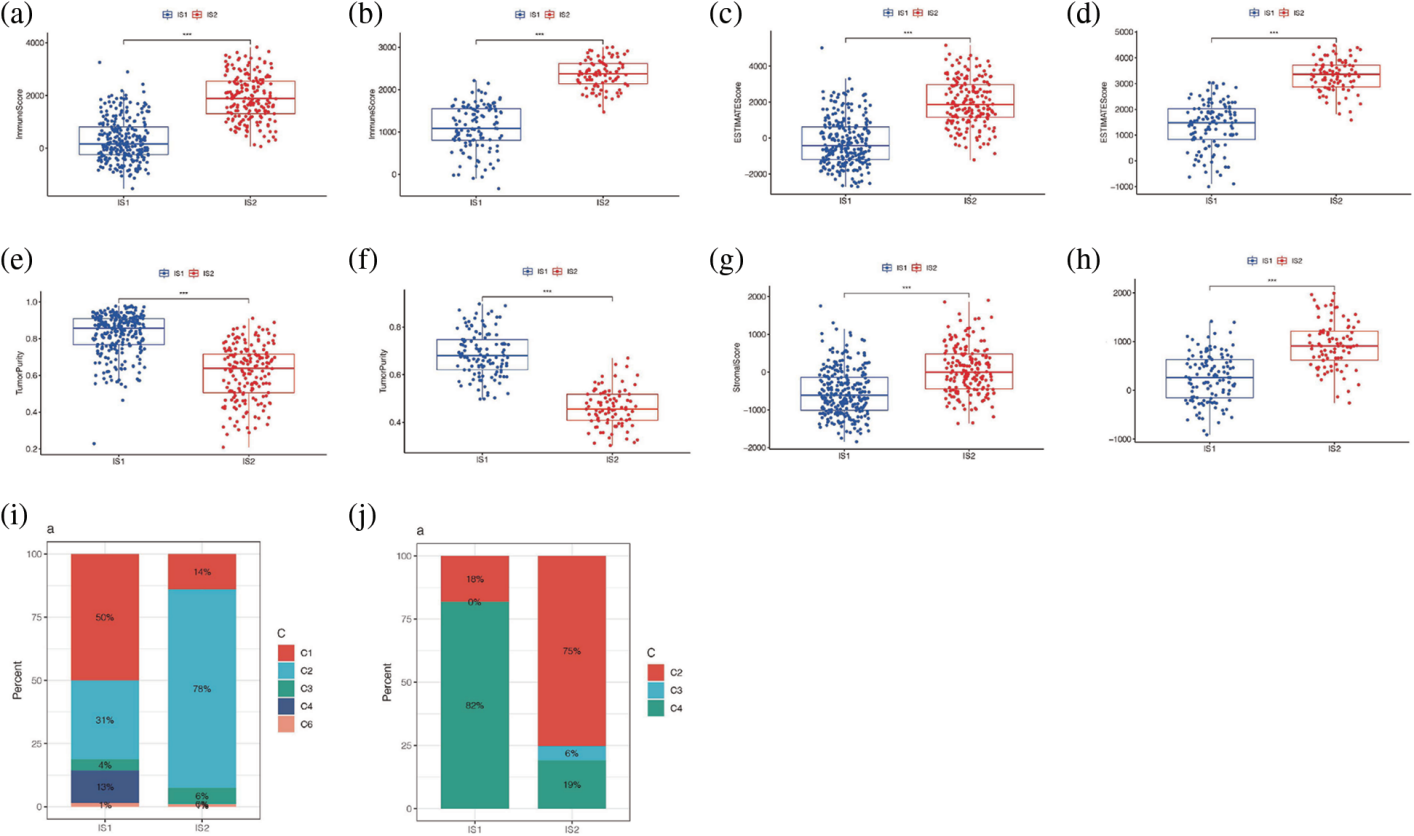
Immune score analysis. (a) Differences in immunity scores between IS1 and IS2 in TCGA cohort. (b) Differences in immunity scores between IS1 and IS2 in the GEO cohort. (c) Difference in ESTIMATE score for IS1 and IS2 in TCGA cohort. (d) The difference between the ESTIMATE scores of IS1 and IS2 in the GEO queue. (e) Differences in tumor purity between IS1 and IS2 in the TCGA cohort. (f) Differences in tumor purity between IS1 and IS2 in the GEO cohort. (g) Differences in STROmal score between IS1 and IS2 in TCGA cohort. (h) Differences in Stromal score between IS1 and IS2 in GEO cohort. (i, j) Matching the results of unsupervised clustering with the known type 6 immune subtypes.

The “ESTIMATE” package further evaluated the immune score and stromal score in the TCGA and GSE65904 cohorts and found that the results were consistent in both cohorts, suggesting that the immune score, stromal score, and total score were higher in the immune subtype IS2 than in the immune subtype IS1 (*p* < 0.001), and the tumor purity of immune subtype IS2 was lower than that of immune subtype IS1 (Figs. 8a–8h). Further, by matching the results of unsupervised clustering with the known type 6 immune subtypes calculated by R package “ImmuneSubtypeClassifier”, it was found that C1 and C2 were predominant in IS1 of TCGA cohort (Fig. 8i), with a higher proportion of C1 than C2. However, in the IS2 subtype, C1 and C2 were still the main components, but the proportion of C2 was significantly higher than that of C1, *p* < 0.05. Then it was found in the GSE65904 cohort (Fig. 8j) that C2 and C4 were the main components in the IS1 subtype, while C2, C3, and C4 were the main components in the IS2 subtype, *p* < 0.05. The results of both cohorts indicated that the main component in the IS2 immune subtype was C2. Combined with the previous research results, it was suggested that there were more immune cells in immune subtype IS2, the immune system was activated, and the tumor tissue level was low. However, the previous analysis found that the prognosis of subtype IS2 was worse than that of IS1, which may indicate that immune subtype IS2 is immune heat type and immune suppression type, while IS1 is immune cold type.

### Immune landscape analysis

**Figure 9:**
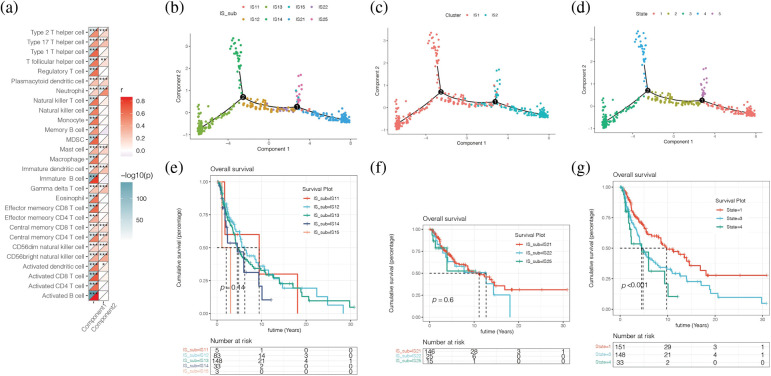
Immune landscape analysis. (a) The correlation between horizontal axis Compontent1 and vertical axis Component2 and the immune cells obtained by ssGSEA analysis. (b) IS1 can be further refined into 5 subtypes, namely IS11, IS12, IS13, IS14, and IS15. IS2 can be further refined into 3 subtypes, IS21, IS22, and IS25. (c, d) IS1 and IS2 had significantly different differentiation states (c), with a total of 5 states (d). (e, f) No significant difference in survival analysis among the refined subtypes within subtype IS1 or IS2. (g) The prognosis of the marginal states such as State1, State3, and State4 was different, among which State1 had a better prognosis.

Fig. 9a showed the correlation between horizontal axis Component1 and vertical axis Component2 in subsequent dimension reduction clustering analysis and immune cells obtained by ssGSEA analysis. By reducing the dimension and conducting immune landscape analysis, it can be found in Fig. 9b that IS1 can be further refined into five subtypes, namely IS11, IS12, IS13, IS14, and IS15. IS2 can be further refined into three subtypes, IS21, IS22, and IS25. The two immune subtypes IS1 and IS2 had significantly different differentiation states (Fig. 9c), with a total of five states (Fig. 9d). We found no significant difference in survival analysis among the refined subtypes within subtype IS1 or IS2 (Figs. 9e and 9f). The prognosis of the marginal states such as State1, State3, and State4 was different, among which State1 had a better prognosis (Fig. 9g). Then we further explored the correlation between horizontal axis Compontent1 and vertical axis Component2 and the immune cells obtained by ssGSEA analysis (Fig. 9a). The results showed that component1 was significantly correlated with 28 kinds of immune cells, such as various immune T cells, B cells, etc. Component2 is associated with 13 kinds of immune cells, including the Type 2 T helper cell, the Type 17 T helper cell, and the Central memory CD8+ T cell, etc.

### Co-expression analysis

**Figure 10:**
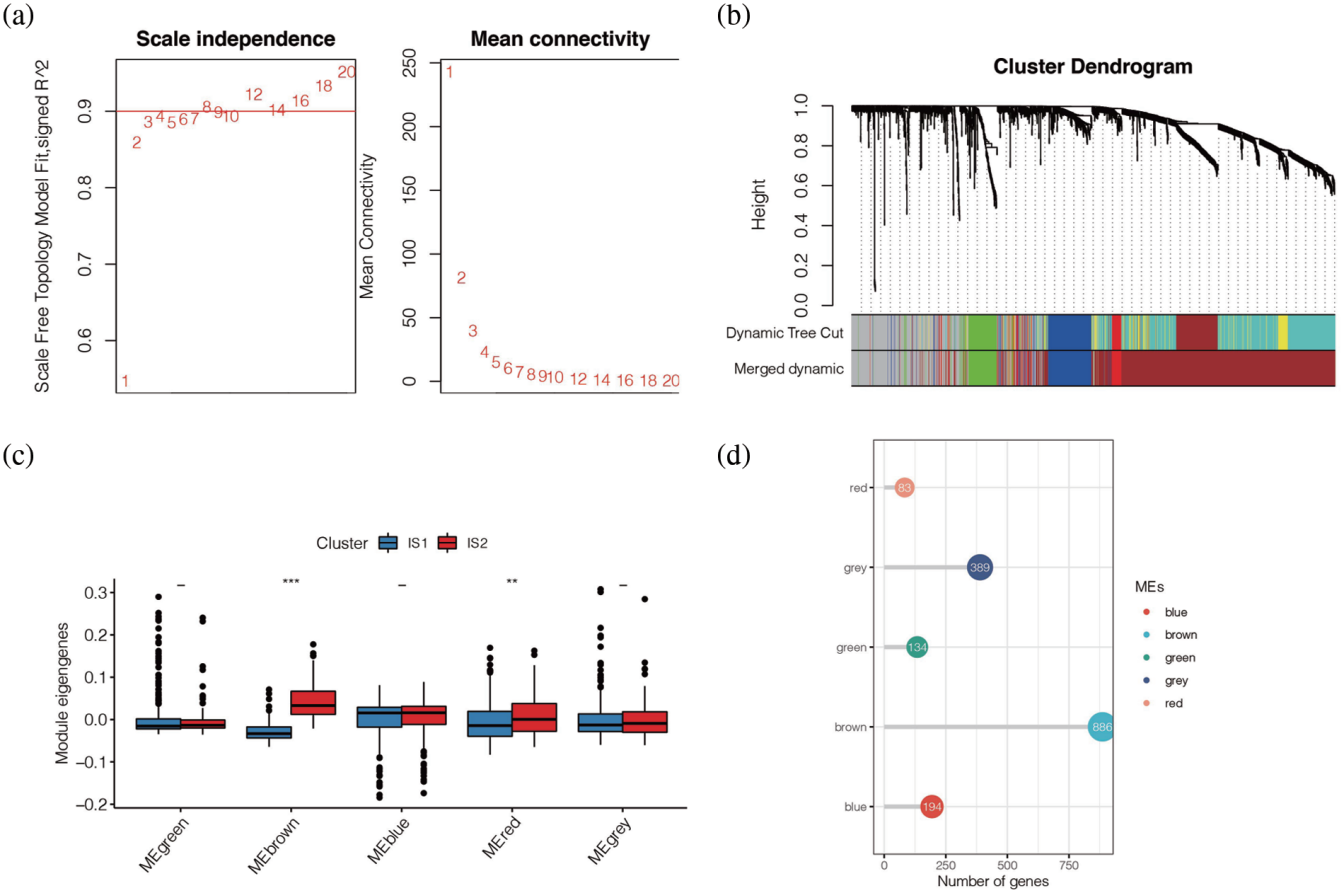
Co-expression analysis. (a) When the soft domain value was 5, R^2 > 0.8, the data was in line with the power-law distribution, and the trend of the graph was flat, which was suitable for subsequent analysis. (b) WGCNA showed that melanoma patients correspond to different modules. (c) Brown and Red modules were highly expressed in IS2. (d) The number of genes in the Brown module was the largest, with 886 co-expressed immune-related genes, while the red module had only 194 immune-related genes.

Subsequently, the study conducted wGCNA co-expression analysis for all immune-related genes obtained and set the optimal soft domain value as 5. As shown in Fig. 10a, when the soft domain value was 5, R^2^ > 0.8, the data was in line with the power-law distribution, and the trend of the graph was flat, which was suitable for subsequent analysis. Then, by setting the minimum number of module genes to 30, deepSplit to 3, and merging modules with MEDissThres lower than 0.3, we end up with 5 modules. In Fig. 10b, we can see that melanoma patients correspond to different modules. To further explore the relationship between these five modules and immune subtypes IS1 and IS2, we conducted a difference analysis, as shown in Fig. 10c, and found that Brown and Red modules were highly expressed in IS2. The number of genes in the Brown module was the largest, with 886 co-expressed immune-related genes, while the red module had only 194 immune-related genes (Fig. 10d).

**Figure 11:**
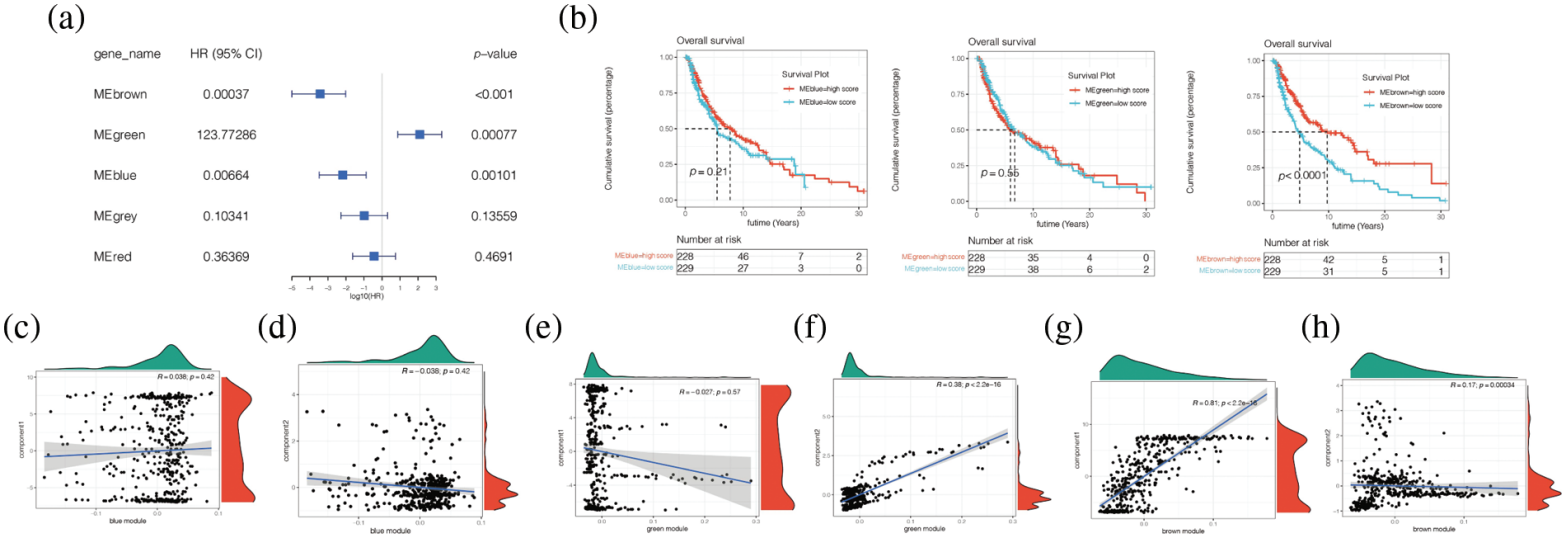
Correlation analysis between different modules and clinical characteristics of patients. (a) Univariate COX analysis found that Brown, Green, and Meblue have an impact on prognosis, and Brown and Meblue are protective factors for melanoma patients, while Green is a risk factor. (b) The survival analysis of these three modules found that the low score group of Brown module had a poor prognosis, while the high score group had a good prognosis (*p* < 0.05), while there was no statistical difference between the survival analysis of Green and Meblue modules. (c–h) Brown module is correlated with ComponerN1 and Component2 (*p* < 0.05). The correlation coefficients were 0.81 and 0.17, respectively.

To investigate whether modules of different colours have differences in patients’ prognoses, univariate COX analysis was carried out and it was found in Fig. 11a that Brown, Green, and Meblue have an impact on prognosis, and Brown and Meblue are protective factors for melanoma patients, while Green is a risk factor. The survival analysis of these three modules found that the low score group of Brown module had a poor prognosis, while the high score group had a good prognosis (*p* < 0.05), while there was no statistical difference between the survival analysis of Green and Meblue modules (Figs. 11a and 11b). Finally, by analysing the correlation between Brown, Green, and Meblue modules and component1 of the horizontal axis and Component2 of the vertical axis (Figs. 11c–11h), we found that the Brown module is correlated with ComponerN1 and Component2 (*p* < 0.05). The correlation coefficients were 0.81 and 0.17, respectively (Figs. 11g and 11h).

### Cell assay verifies the role of IDO1 in melanoma cell line A375 and WM-115 in vitro

**Figure 12:**
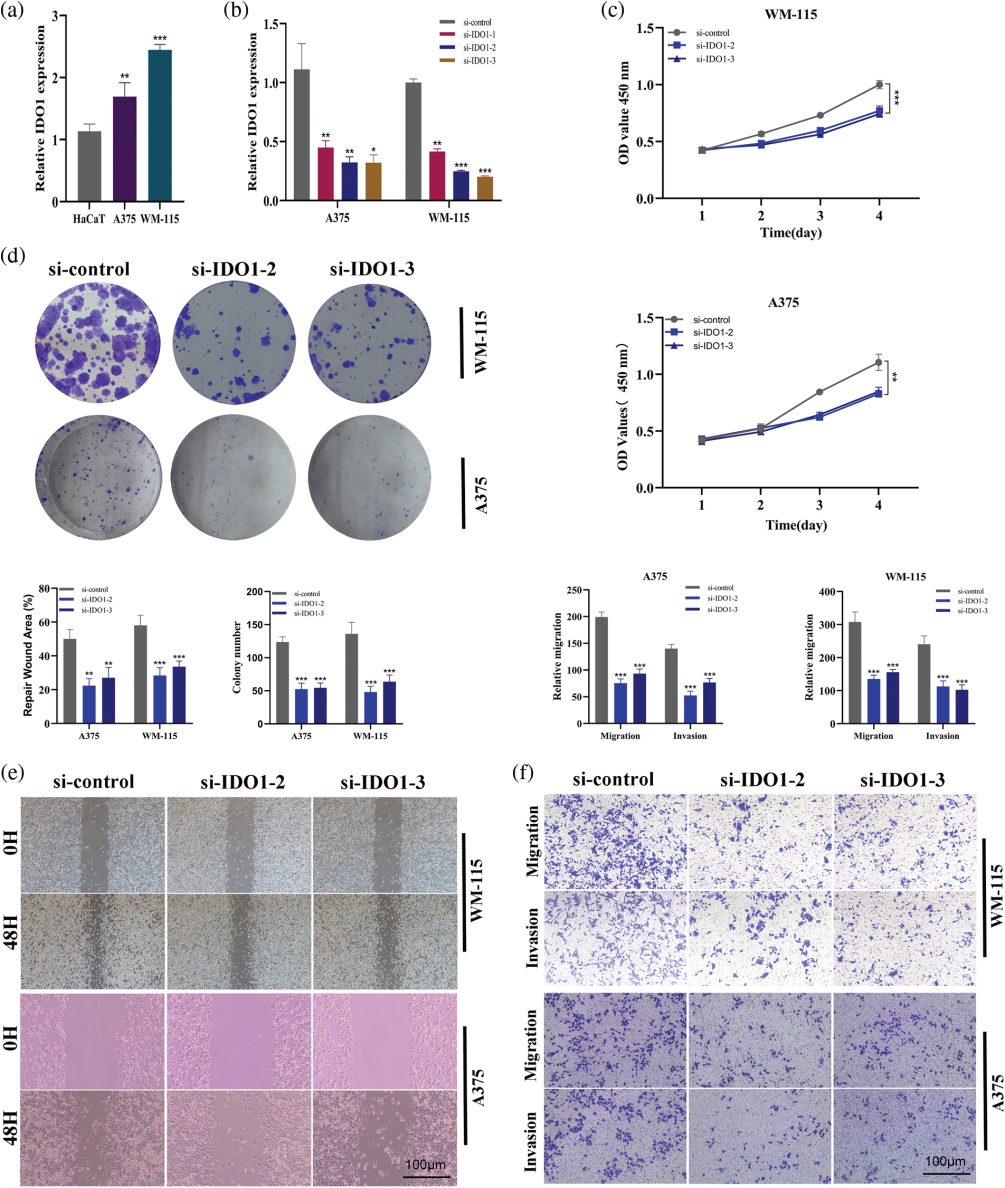
Cell assay verifies the role of IDO1 in melanoma cell line A375 *in vitro*. (a) PCR results showed that IDO1 expression was up-regulated in melanoma A375 cell lines compared with normal epidermal HaCaT cell lines (*p < 0.05). (b) The A375 cell line was transfected with three siRNAs and PCR results showed that all three siRNAs significantly knocked down IDO1 expression, with higher knockdown efficiency in si-IDO1-2 and si-IDO1-3 (*p < 0.05; **p < 0.01). (c) CCK-8 assays. The cell viability of A375 cell lines was decreased significantly after IDO1 knockdown (**p < 0.01). (d) Clone formation experiments. The proliferation ability of A375 cells decreased significantly after IDO1 knockdown. (e) Transwell experiments. The migration and invasion abilities of A375 cell lines were significantly reduced after IDO1 knockdown (**p < 0.01, ***p < 0.001). (f) Scratch experiment. The migration rate of A375 cell line decreased significantly after IDO1 knockdown (**p < 0.01). All of our experiments were repeated three times.

First, PCR results showed that IDO1 expression was upregulated in melanoma A375 cell lines and WM-115 cell lines compared with normal epidermal HaCaT cell lines (Fig. 12a, *p < 0.05). Subsequently, the A375 cell line and WM-115 cell lines were transfected with three siRNAs and PCR results showed that all three siRNAs significantly knocked down IDO1 expression, with higher knockdown efficiency in si-IDO1-2 and si-IDO1-3 (Fig. 12b, *p < 0.05; **p < 0.01). So subsequent functional experiments were performed in the SI-IDO1-2 and SI-IDO1-3 groups. CCK-8 showed a significant decrease in cell viability of A375 cell lines and WM-115 cell lines after IDO1 knockdown (Fig. 12c, **p < 0.01). Clone formation experiments showed that the proliferation ability of A375 cells and WM-115 cell lines decreased significantly after IDO1 knockdown (Fig. 12d, **p < 0.01). A scratch experiment showed that migration rate of A375 cell line and WM-115 cell lines decreased significantly after IDO1 knockdown (Fig. 12e, **p < 0.01). Transwell experiments showed that the migration and invasion abilities of A375 cell lines and WM-115 cell lines were significantly reduced after IDO1 knockdown (Fig. 12f, **p < 0.01, ***p < 0.001). Considering that our survival analysis shows that IDO1 is highly expressed in human melanoma with a good prognosis, the role of IDO1 *in vivo* may have a more complex mechanism, such as activation of the immune system. All of our experiments were repeated three times.

## Discussion

As the most malignant skin tumor, skin cutaneous melanoma still has a poor prognosis [15]. In the immune microenvironment of melanoma, cytotoxic T-lymphocyte-associated protein-4 and programmed cell death protein-1 on the T-cell membrane bind to ligands on dendritic cells, thereby attenuating tumor immunity and leading to tumor progression [16]. Currently, immune checkpoint inhibitors based on CTLA-4 and PD-1 have been approved for the immunotherapy of melanoma [17]. Therefore, antigen-presenting cells, especially dendritic cells, play an important role in tumor immunity and immunotherapy [18]. Many current mRNA vaccines are also associated with dendritic cells [19]. By introducing mRNA encoding tumor antigens into dendritic cells, the dendritic cells can express tumor antigens, thereby activating immune responses and treating tumors [20]. Some studies have found that the combination of mRNA vaccine and immune checkpoint inhibitor can improve treatment response [21]. Therefore, the mRNA vaccine is a promising treatment. Screening for meaningful tumor antigen-coding mRNAs can improve patient outcomes [22].

In this study, we identified potential melanoma tumor antigens: GZMB, GBP4, CD79A, APOBEC3F, IDO1, JCHAIN, LAG3, PLA2G2D, XCL2 by mutation analysis, expression analysis, and immune infiltration correlation analysis. We then downloaded a cohort of melanoma patients from a public database to identify immune subtypes of melanoma suitable for vaccination. First, we divided melanoma patients into IS1 and IS2 subtypes by unsupervised cluster analysis. Subsequently, mutation landscape analysis, immune correlation analysis, immune infiltration analysis, immune landscape analysis, and weighted co-expression network analysis proved that there were differences between IS1 and IS2 in tumor immunity. IS2 was the immune-hot type, while IS1 was the immune-cold type.

Tumor antigens include a tumor-specific antigen (TSA) and tumor-associated antigen (TAA) [23]. TSA is expressed only in tumors and usually results from genetic mutations [24]. TAA expression was low in normal tissues but upregulated in tumors [25]. The high cost of TSA testing has hindered its development in mRNA vaccine research [23]. Therefore, the TAA vaccine is the main mRNA vaccine widely developed at present. We identified potential tumor-associated antigens in melanoma patients: GZMB, GBP4, CD79A, APOBEC3F, IDO1, JCHAIN, LAG3, PLA2G2D, XCL2. GZMB is a Granzyme subfamily of proteins associated with chronic inflammation and wound healing [26]. GBP4 protein is a kind of guanosine binding protein, which is related to cytokine signal transduction in the immune system and has GTP enzyme activity [27]. CD79A protein is involved in antigen-activated B cell receptor signalling pathways [28]. Apobec3f-related research has focused on immune system diseases, especially immunodeficiency diseases [29]. Current studies have shown that IDO1 is highly correlated with glucose metabolism and amino acid metabolism [30]. JCHAIN is involved mainly through the binding of scavenger receptors and ligands [31]. LAG3 protein is mainly involved in the antigen presentation of MHCII molecules and the antigen processing and presentation of MHCII molecules [32]. The pathways involved by PLA2G2D include PE acyl chain remodelling and oxytocin signalling [33]. XCL2 is mainly involved in the activation pathway of the chemokine superfamily [34]. In this study, we found these genes are upregulated in melanoma and are associated with improved prognosis. Further studies found that these genes were highly correlated with immune cell infiltration, suggesting that the improvement of melanoma prognosis may be associated with tumor immunity. This cannot only provide a reference for the diagnosis and treatment of melanoma patients to a certain extent but also deepen our understanding of the role of these genes in melanoma.

Immune reprogramming is widely found in various tumors, especially melanoma, which is highly immune-related [35]. Due to genomic instability, there is significant heterogeneity between patients [36]. Therefore, not only do we need to identify potential tumor antigens, but we also need to do immunotyping of melanoma patients to identify people who are suitable for vaccination. Our study found that melanoma patients can be divided into IS1 and IS2 subtypes. There were significant differences in prognosis, mutation landscape, immune infiltration, and immune landscape between the two subtypes. This undoubtedly provides a reference for the treatment and prognosis of melanoma.

GSE65904 is the dataset used in our study. In the original paper on this dataset, the authors used gene-expression profiling to molecularly stratify metastatic melanoma and found an increased risk of developing distant metastases in the pigmentation and proliferative groups compared with the hyperimmunoreactive group [37]. In our study, we provide potential tumor antigens for vaccine development in melanoma patients, and we also divide melanoma patients into two immune subtypes, which have significant differences in tumor immunity and may have different responses to vaccination.

The tumor antigens and immunological subtypes of various malignancies have currently been identified by numerous research. By examining GEO and TCGA data, Huang et al. discovered three tumor antigens in cholangiocarcinoma, namely CD247, FCGR1A, and TRRAP. Additionally, they separated cholangiocarcinoma patients into immune “hot” and immune “cool” kinds, which had noticeably different results [38]. A total of six tumor antigens, including ADAM9, EFNB2, MET, TMOD3, TPX2, and WNT7A, were also discovered by Huang et al. in pancreatic cancer [39]. Additionally, they divided pancreatic cancer into FIVE different immunological subtypes, each of which had a specific immune state and prognosis. In our study, we identified GZMB, GBP4, CD79A, APOBEC3F, IDO1, JCHAIN, LAG3, PLA2G2D and XCL2 as potential tumor antigens of melanoma by multi-omics analysis, and divided melanoma into two different immune subtypes. Our study provides a reference for precision treatment and immunotherapy of melanoma. However, there are some limitations to our study. We lack a clinical cohort to verify our results, which we will improve in the future.

## Conclusion

We identified potential tumor antigens for cutaneous melanoma using a combination of multiple assayed methods. In addition, we divided melanoma patients into two immune subtypes, which have significant differences in prognosis and immunity, which may provide reference for the appropriate population for vaccination.

## Data Availability

All the data can be found in the manuscript.

## Supplementary Materials

**Supplemental Table S1:** Sequence of the siRNA

Gene name	Sequence	
	Sense (5′-3′)	Antisense (5′-3′)
IDO1-homo-1033	GUGCUCAUUAGAGUCAAAUTT	AUUUGACUCUAAUGAGCACTT
IDO1-homo-915	GCGUCUUUCAGUGCUUUGATT	UCAAAGCACUGAAAGACGCTT
IDO1-homo-133	CUCCUGGACAAUCAGUAAATT	UUUACUGAUUGUCCAGGAGTT

**Supplemental Table S2:** Primer sequences for PCR experiments

IDO1 (Forward)	AAGCCCCTGACTTATGAGAACA
IDO1 (Reverse)	GCGCCTTTAGCAAAGTGTCC
β-actin (Forward)	TCAAGATCATTGCTCCTCCTGAG
β-actin (Reverse)	ACATCTGCTGGAAGGTGGACA
